# Dietary Variation and Evolution of Gene Copy Number among Dog Breeds

**DOI:** 10.1371/journal.pone.0148899

**Published:** 2016-02-10

**Authors:** Taylor Reiter, Evelyn Jagoda, Terence D. Capellini

**Affiliations:** Human Evolutionary Biology, Harvard University, 11 Divinity Avenue, Cambridge, MA, 02138, United States of America; CSIRO, AUSTRALIA

## Abstract

Prolonged human interactions and artificial selection have influenced the genotypic and phenotypic diversity among dog breeds. Because humans and dogs occupy diverse habitats, ecological contexts have likely contributed to breed-specific positive selection. Prior to the advent of modern dog-feeding practices, there was likely substantial variation in dietary landscapes among disparate dog breeds. As such, we investigated one type of genetic variant, copy number variation, in three metabolic genes: *glucokinase regulatory protein* (*GCKR*), *phytanol-CoA 2-hydroxylase* (*PHYH*), and *pancreatic α-amylase 2B* (*AMY2B*). These genes code for proteins that are responsible for metabolizing dietary products that originate from distinctly different food types: sugar, meat, and starch, respectively. After surveying copy number variation among dogs with diverse dietary histories, we found no correlation between diet and positive selection in either *GCKR* or *PHYH*. Although it has been previously demonstrated that dogs experienced a copy number increase in *AMY2B* relative to wolves during or after the dog domestication process, we demonstrate that positive selection continued to act on amylase copy number in dog breeds that consumed starch-rich diets in time periods after domestication. Furthermore, we found that introgression with wolves is not responsible for deterioration of positive selection on *AMY2B* among diverse dog breeds. Together, this supports the hypothesis that the amylase copy number expansion is found universally in dogs.

## Introduction

The remarkable phenotypic diversity amongst dog breeds has been affected in part by intensive and prolonged human-facilitated artificial selection since the initial period of dog domestication [1]. Following domestication, human influence has exerted a selection pressure, which, alongside natural selection and stochastic genetic processes such as genetic drift [2], shaped dog biology. For example, metabolic adaptations to new diets arose in dog breeds spanning geographic and climatic ranges that mirror the cohabitation patterns of humans [3]. In this context, selective pressures directly and indirectly imposed by humans likely impacted the evolution of canine biology by leading to the emergence and persistence of positively-selected traits over the course of human and dog coevolution. On the other hand, stochastic evolutionary forces such as drift and bottlenecks may have changed the trajectory of positive selection in canines during human and dog co-migration.

Though the exact timespan and location of initial dog-human cohabitation and domestication remains unclear, humans and dogs have lived amongst one another for thousands of years [4–6]. Consensus from fossil and genetic evidence places the lower bound date of domestication around 14,000 BP [7–10]. While most genetic research supports a single center of domestication [7,9,11,12], the location of this center is contentiously debated.

Although the driving forces of dog domestication by humans remains unclear, dogs have nonetheless evolved suites of dietary and metabolic adaptations that have been argued to reflect their direct interaction with humans. It has been suggested that humans provided food for local dogs both directly and indirectly throughout dog-human coevolution [13]. For example, historically, it was believed that dogs were fed directly by their caretakers from human provisions [14]. However, more recently it has been argued that initially humans may have indirectly fed dogs by creating food waste from which wild canids scavenged [13,15]. Regardless of intention, this (direct or indirect) provisioning may have acted as a defining selective agent in the dog domestication process [13,15], with important impacts on canine metabolism.

Over time, and via cohabitation, the canine diet has been transformed from the carnivorous diet of its ancestor, the wolf, to a diet more closely matching that of omnivorous humans [13]. This transformation increased variation in the domesticated dog’s diet, potentiating impact on numerous biological pathways. This increase in variation was extreme in some cases: isotopic analysis has shown that by 7000 BC in northern China, 65–90% of domesticated dogs’ diets were comprised of millet alone [5]. However, the onset and sources of dietary variation were not universal—in 3500–2000 BC, dogs living among Korean shell midden cultures received the majority of their calories from marine mammals and other fish [16]. Because humans populated a variety of habitats with different dietary staples, dog breeds from different places also consumed diets composed of unique combinations of food items. For many breeds, dietary changes resulted in increases in novel food constituents that may have required new, better, or more digestive mechanisms, thereby exerting differential selective forces on dogs living among different groups of humans. For example, starch digestion presented a new dietary challenge to which the dog likely adapted through alteration of three key genes in the starch digestion pathway [15]. Other novel dietary changes for certain breeds likely include high intake of marine mammals and fish [17,18].

Selection on variability in both single-base pair and copy number variants may underlie key metabolic changes in the domesticated dog. Interestingly, in instances of prolonged dog-human cohabitation, there is evidence of parallel evolution in metabolic genes. For example, compared to native Chinese dogs, Tibetan mastiffs have 33 SNPs in 11 genes that display signatures of positive selection, the majority of which cluster in 9 genes related to high-altitude adaptations [19]. Likewise, Tibetans have experienced selective sweeps for haplotypes in genes involved in high-altitude adaptations some of which are likely the introduced through interbreeding with archaic humans [20]. More pertinently, amylase genes responsible for digesting starch have undergone copy number duplications in both dogs and humans [15,21]. These duplications translate to a functional increase in the ability to digest starches into sugar [21–23].

The goal of this study was to investigate how potential selection pressures created by exposure to different diets affected copy number variation (CNV) in six dog breeds from diverse dietary backgrounds: Pekingese, Shar Pei, Shiba Inu, Akita, Siberian Husky, and Alaskan Malamute. The Pekingese and Shar Pei likely consumed high-starch diets [5,24–26], while the Akita and Shiba Inu (Japanese dogs), Siberian Husky, and Alaskan Malamute likely consumed low-starch, seafood-rich diets [27–30]. We specifically focused on three metabolic and dietary genes: *glucokinase regulator* (*GCKR*), which plays a key role in maintaining blood glucose homeostasis; *phytanol CoA 2-hydroxylase* (*PHYH*), which codes for the protein responsible for the first step of phytanic acid digestion [31] often found in ruminant meat and seafood [32]; and *pancreatic α-amylase 2B* (*AMY2B*), which, as discussed above, is involved in the starch digestion.

By further investigating copy number variation in the *AMY2B* gene of dog breeds with both starch-rich and starch-poor diets, we were able to explore the relationship between diet and copy number post-domestication when diet likely depended greatly on location and human interactions. We found evidence that positive selection continued to affect *AMY2B* CNV in dog breeds that consumed starch-rich diets, but that genetic drift affected *AMY2B* CNV more in dogs that began consuming starch-poor diets. The data presented in our study support early positive selection on increased *AMY2B* CNV across all breeds of domesticated dogs.

## Results

### Diet does not predict *GCKR* CNV or *PHYH* CNV

**Fig 1:**
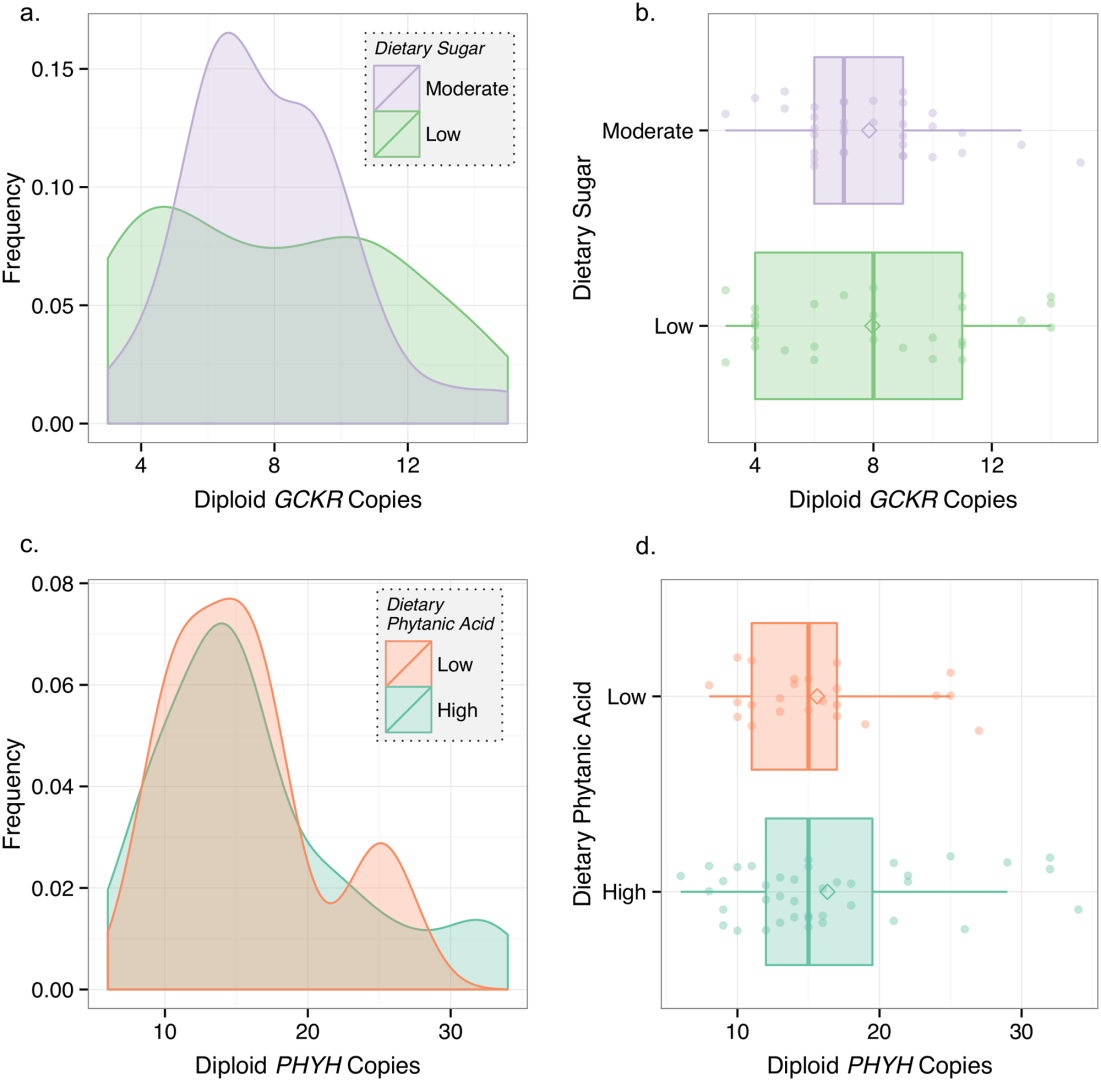
Diet and *GCKR* and *PHYH* copy number variation. **a**) Density plot of ddPCR diploid *GCKR* copy number for dogs that traditionally consumed high sugar diets and low sugar diets. Density reflects frequency with which a given diploid copy number appears in each population. **b**) Tukey boxplot of diploid *GCKR* copy number for dogs that traditionally consumed high sugar diets and low sugar diets. **c**) Density plot of ddPCR diploid *PHYH* copy number for dogs that traditionally consumed high phytanic acid diets and low phytanic acid diets. **d**) Tukey boxplot of diploid *PHYH* copy number for dogs that traditionally consumed high phytanic acid diets and low phytanic acid diets.

To investigate the impact of diet on copy number variation in *GCKR*, *PHYH*, and *AMY2B*, we determined a crude dietary composition by probing archaeological and ethnographic records (Methods). We also determined the copy number of each individual in each gene using digital droplet PCR (Methods). We found that sugar intake did not predict *GCKR* copy number across breeds. Specifically, dogs with moderate sugar intake (Shar Pei, Pekingese, and Japanese dogs; n = 34) had a mean CNV of 8.1±3.6, while dogs with low sugar intake (Siberian Husky and Alaskan Malamute; n = 28) had a mean CNV of 7.8±2.5 (Robust rank order test: p = .98; Fligner-Killeen test: p = .01) (Fig 1). Although our proxies indicate that the Alaskan Malamute and Siberian Husky consumed the least sugar of the six dog breeds, the averages of these dog breeds fell at disparate ends of the breed-wide distribution.

Because of its role in regulating blood glucose homeostasis, it has previously been suggested that *GCKR* copy number may play a role in diabetes incidence in some dog breeds [33]. To investigate this further, diploid *GCKR* CNV was compared to diabetes risk for breeds for which this information was available (Pekingese, Siberian Husky, and Alaskan Malamute). Two breeds, the Siberian Husky and the Pekingese, both had duplications relative to the reference sequence CanFam2. However, the diabetes risk for these two breeds was not the same, with Pekingese having lower risk and the Siberian Husky having higher risk. In addition, the Alaskan Malamute, which experienced both gains and losses relative to CanFam2, displayed no evidence of increased risk for diabetes (S1 Table).

We next investigated the relationship between our proxies of dietary intake of phytanic acid and *PHYH* copy number, and similarly found no relationship; intake did not predict *PHYH* copy number. Specifically, the mean CNV of dogs that consumed high phytanic acid diets (Alaskan Malamute, Siberian Husky, and Japanese dogs; n = 39), 16.2±7.1 was not significantly different from that of dogs that consumed low phytanic acid diets (Pekingese, and Shar Pei; n = 23) which had a mean CNV of 15.5±5.4 (Robust rank order test: p = .92; Fligner-Killeen test: p = .48) (Fig 1). Though these results preliminarily suggest that dietary intake does not correlate with copy number variation in these genes, it is important to note that ddPCR has limited power to accurately predict copy number at high numbers of duplications (Discussion).

### Starch intake predicts diploid *AMY2B* CNV in some dog breeds

**Fig 2:**
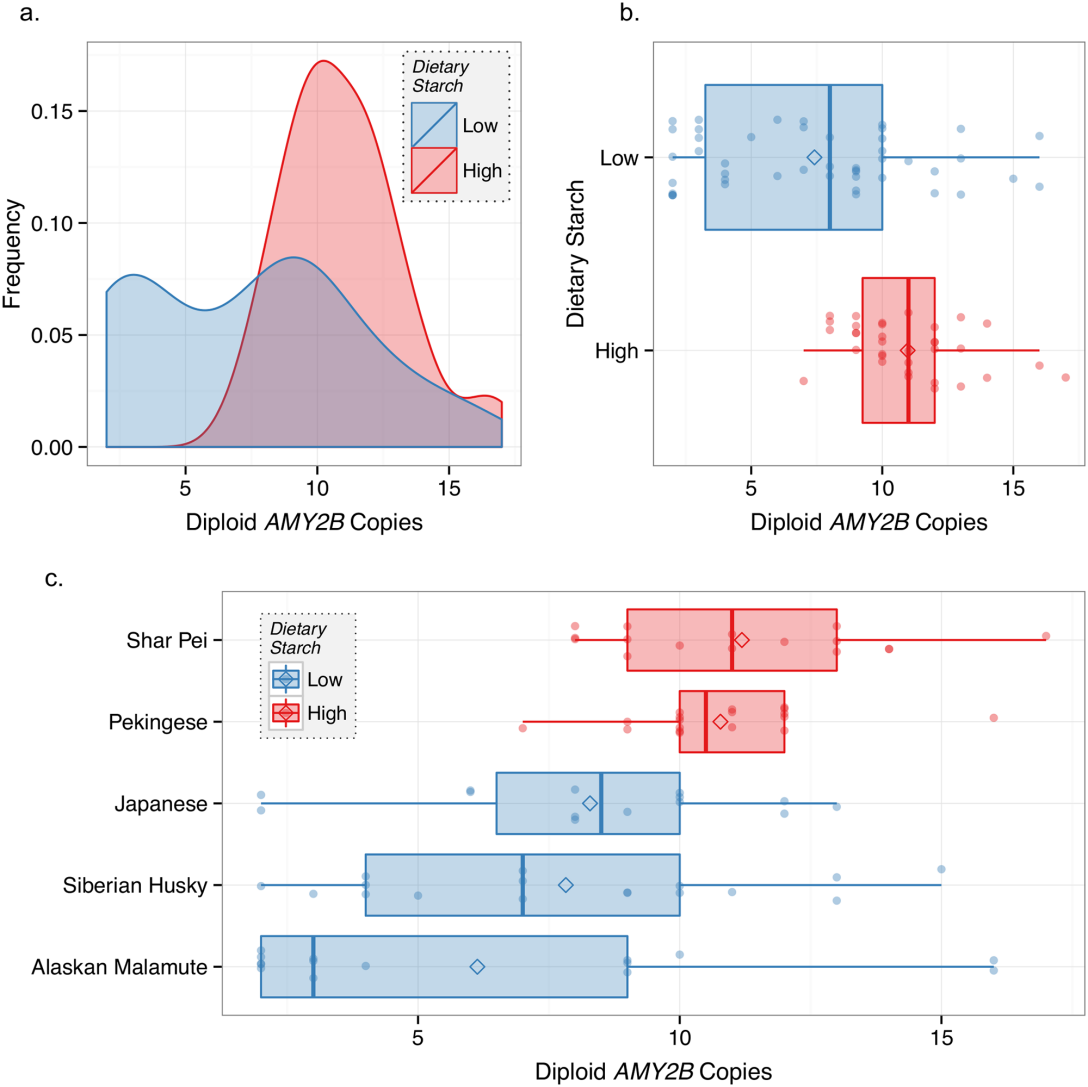
Diet and *AMY2B* copy number variation. **a**) Density plot of ddPCR diploid *AMY2B* copy number for dogs that traditionally consumed high-starch diets and low-starch diets. Density reflects frequency with which a given diploid copy number appears in each population. **b**) Tukey boxplot of diploid *AMY2B* copy number for dogs that traditionally consumed high-starch diets and low-starch diets. **c**) Tukey boxplot of diploid *AMY2B* copy number for specific dog breeds that traditionally consumed high-starch diets and low-starch diets.

Given the history of studies on the role of amylase CNV and starch digestion in humans [21] and dogs [15,22], we next examined *AMY2B* copy number in our expanded dataset, which includes dog breeds representing localities with historically low levels of dietary starch intake (Methods). Consistent with studies with more limited datasets, we found that *AMY2B* CNV did vary with dietary starch intake. Dogs with high-starch diets (Pekingese and Shar Pei; n = 34) had a statistically significant higher mean CNV of 10.9±2.2, compared to dogs with low-starch diets (Siberian Husky, Alaskan Malamute, and Japanese dogs; n = 46) with a mean CNV of 7.4±4.2 (Robust rank order test: p < .0001; Fligner-Killeen test: p = .0004) (Fig 2, S1 Appendix). Importantly, we identified the same significant relationship regardless if we used aggregated data (n = 80), and/or collected samples (n = 62) (Robust rank order test: p = .0002; Fligner-Killeen test: p = .001). The proportion of dogs from combined high starch consuming breeds with at least 10 *AMY2B* copies (74%) was more than two times greater than that for the low starch consuming dogs (33%).

We next compared *AMY2B* CNV within breeds and found that *AMY2B* copy number also differed between high and low starch consuming dogs (Table 1). For example, the Shar Pei (n = 16) had a mean CNV of 11.1±2.7, the Pekingese (n = 18) of 10.7±1.7, the Japanese dogs (n = 14) of 8.3±3.4, the Siberian Husky (n = 17) of 7.8±3.9, and the Alaskan Malamute (n = 15) of 6.1±5.0 (Fig 2). Importantly, dog breeds that consumed high-starch diets were also more similar to each other both in mean copy number and in variation of copy number than to dog breeds that consumed low-starch diets. Breeds that consumed low-starch diets had markedly higher variation in copy number and lower average copy number than breeds that consumed high-starch diets. This could be evidence of positive selection, however we cannot rule out the possibility that stochastic forces led to this pattern from ddPCR data alone.

**Table 1:** Average diploid *AMY2B* copy number and number of samples taken for specific breeds.

Breed	Samples	Starch Intake	Avg Diploid *AMY2B* Copy Number
Chinese Shar Pei	16	High	11.1±2.7
Pekingese	18	High	10.7±1.7
Japanese Dogs	14	Low	8.3±3.4
Siberian Husky	17	Low	7.8±3.9
Alaskan Malamute	15	Low	6.1±5.0

### Diploid *AMY2B* CNV differs from other copy number variable regions

Although our ddPCR data preliminarily suggest and build upon previous suppositions relating to positive selection acting on *AMY2B* copy number, such a selective signature cannot be detected directly from diploid copy number alone. Because traditional methods of identifying positive selection cannot be employed for analysis of diploid copy number, we acquired array-based comparative genomic hybridization data (aCGH) for the Alaskan Malamute and Shar Pei in order to compare patterns in *AMY2B* CNV to genome-wide patterns in CNV. This comparison allowed us to investigate if positive selection acted on *AMY2B* copy number differently in dog breeds with disparate starch intake. Previously, aCGH data from copy number variable sites was compiled for four Alaskan Malamutes and five Shar Pei dogs [34] (GEO http://www.ncbi.nlm.nih.gov/geo/ accession number GSE26170). This distribution was used to compare the extent of breed-level differentiation at the *AMY2B* locus to other genomic loci for the Alaskan Malamute and the Shar Pei.

**Fig 3:**
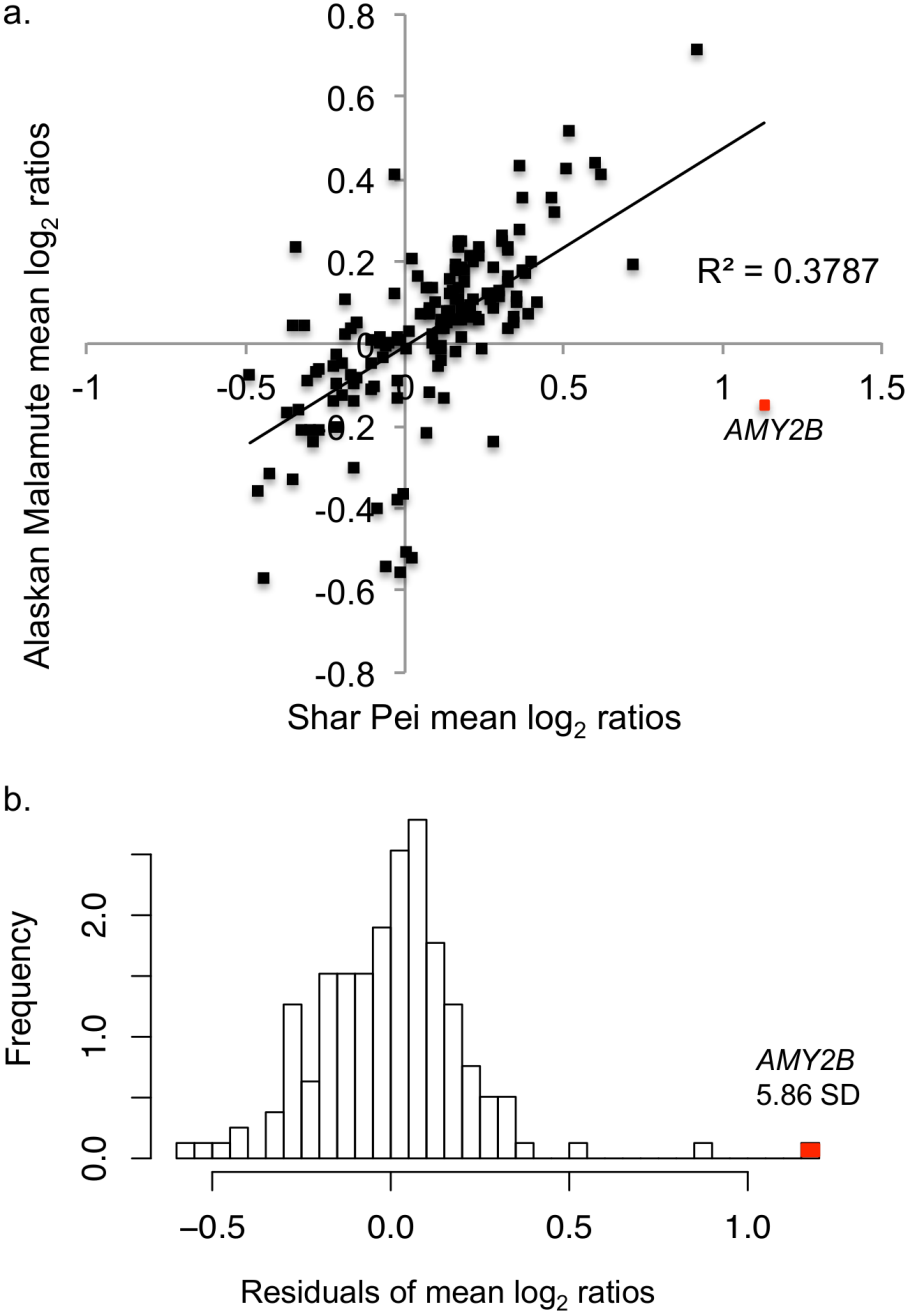
Results from analysis of aCGH data. **a**) Relationship between Shar Pei and Alaskan Malamute mean log_2_ ratios for 157 copy number variable sites in the dog genome. *AMY2B* mean log_2_ ratio is plotted in red, and deviates significantly from the null distribution. **b**) Histogram of residuals from linear regression performed on mean log_2_ ratios from aCGH data in the Alaskan Malamute and Shar Pei. Value of residuals appears on the x-axis while density, or proportion of a specific residual occurring, falls on the y-axis. The residual for mean log_2_ ratio in *AMY2B* is indicated in red and falls 5.86 standard deviations away from the mean (0±.2).

To determine if *AMY2B* CNV mean log_2_ ratio (Methods) deviated substantially from the null distribution of other copy number variable sites in the genome, we analyzed residuals of *AMY2B* to a linear regression line fit to the aCGH data. We found that *AMY2B* fell 5.8 standard deviations away from the mean, indicating that in one or both breeds, *AMY2B* has experienced unusual evolutionary pressures dissimilar to those experienced at other copy number variable sites throughout the genome (Fig 3). However, because aCGH data was only available for nine dogs, this analysis was supplemented by comparisons generated by 55 samples from 10 other breeds: Border Collie, Beagle, Brittany Spaniel, Boxer, Dachshund, Greyhound, German Shepard, Jack Russell Terrier, Labrador Retriever, and Standard Poodle (S2 Appendix). When compared to other null distributions of copy number variation, *AMY2B* fell 15 standard deviations away from the mean.

When these results are supplemented by data obtained by ddPCR, these patterns support that the Shar Pei continued to experience positive selection at *AMY2B* after the initial duplication event, while *AMY2B* CNV in the Alaskan Malamute may have been subject to genetic drift or weak negative selection.

### Starch intake predicts species-wide differences in diploid *AMY2B* CNV

Prior to the adoption and global dominion of agriculture, human starch intake dramatically decreased above 40° latitude [35,36]. With the beginning of agriculture, archaeological accounts indicate that all domestication centers were below 40° latitude and agriculture then spread more slowly on a north-south axis than on an east-west axis [37]. Because starch intake was prehistorically and historically high below 40° latitude and low above 40° latitude, we used latitude as a proxy for starch intake in line with several other studies [35,36].

**Fig 4:**
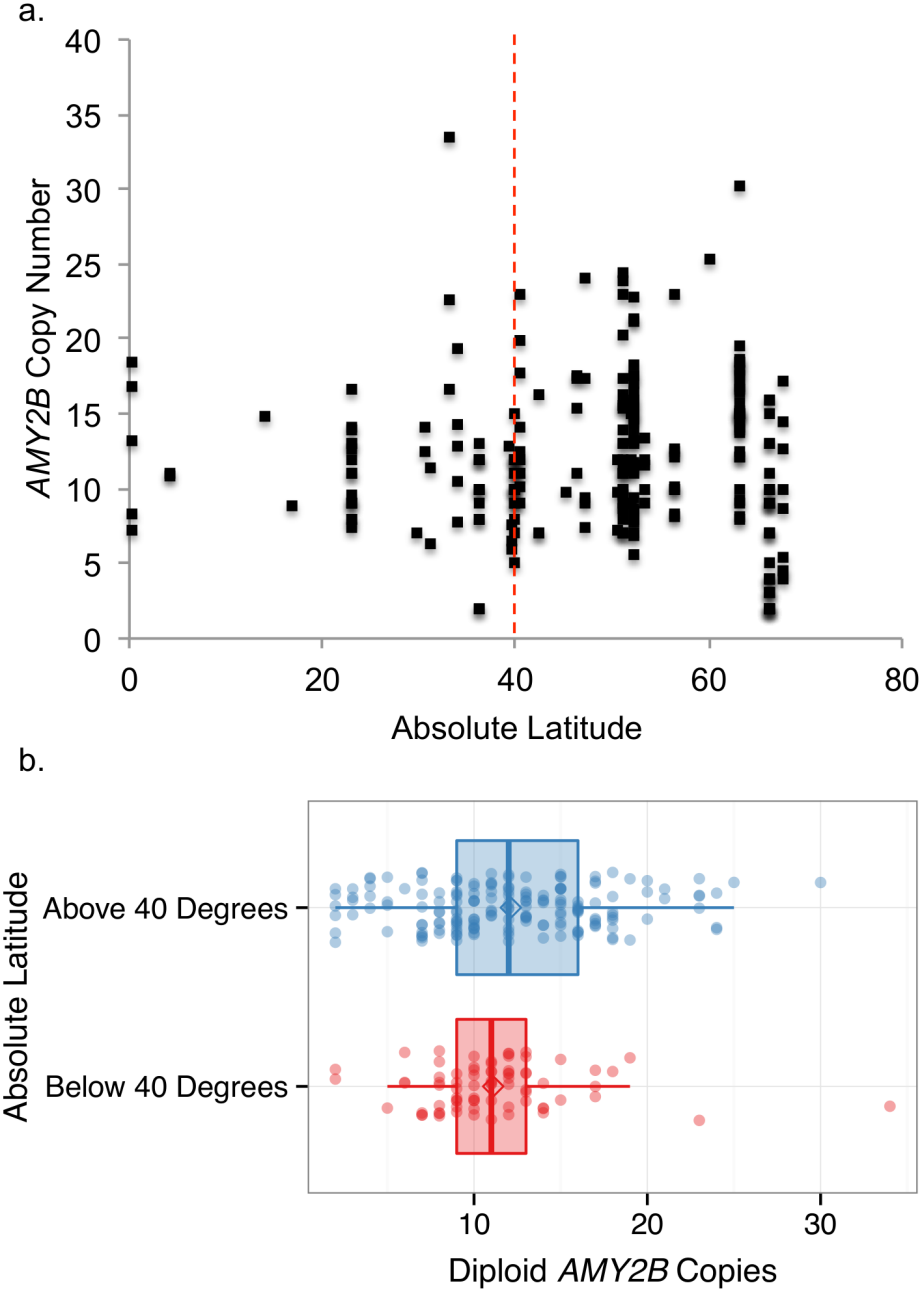
Absolute latitude and starch intake. **a**) *AMY2B* CNV regressed by absolute latitude at breed location of origin for 252 dogs. When absolute latitude is used as a proxy for starch intake, absolute latitude does not predict *AMY2B* copy number. Variation increases at 40° latitude. **b**) Tukey boxplot of *AMY2B* copy number in dog breeds originating from below 40° latitude and above 40° latitude. Variation in *AMY2B* copy number increases at higher latitudes.

We found that absolute latitude did not predict copy number (R^2^ = .003; p = .4). However, variation in *AMY2B* CNV increased with increasing absolute latitude (Fig 4) such that variation in CNV calls from below 40° latitude (n = 75; mean = 11.1±4.4) was statistically different from the variation in CNV calls from above 40° latitude (n = 177; mean = 12.1±5.3) (Fligner-Killeen: p < .001) (Fig 4). As illustrated with ddPCR data, variation in *AMY2B* copy number increases as starch consumption declines, but average *AMY2B* copy number is universally high in all dog breeds compared to wolves [15].

### Phylogeny does not predict *AMY2B* CNV between breeds

**Fig 5:**
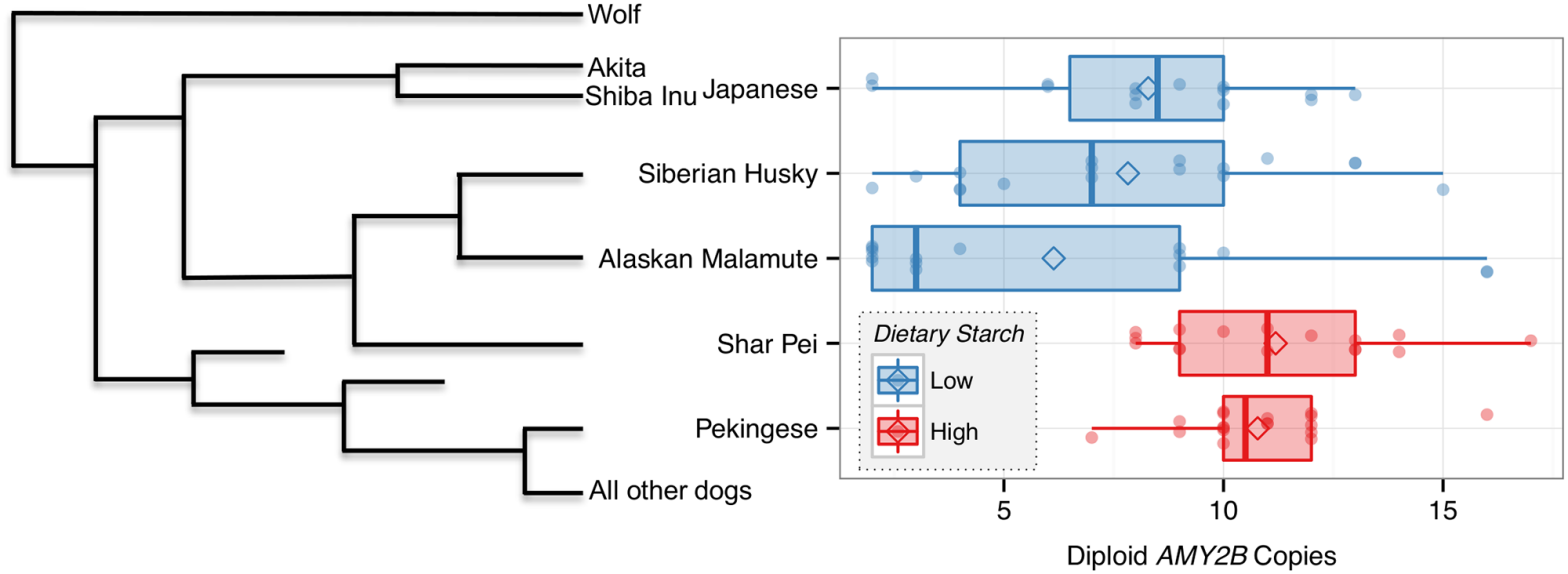
Cladogram depicting the relationship between the dog breeds evaluated in this study and diploid *AMY2B* copy number. Cladogram relations were determined from SNP data and was adapted from vonHoldt et al. [12].

In order to determine whether the above patterns in mean CNV or CNV variation reflect some historical (artificial) selection signal or random forces, we sought to determine if phylogeny played a role. Given the limited statistical power to detect phylogenetic signal using a dataset consisting of only six dog breeds, phylogeny could not be controlled for with traditional methods [38]. Instead, to gauge how phylogenetic signals could potentially explain our findings we mapped *AMY2B* CNV onto two different cladograms [12,39], each of which depicted the phylogenetic relationship between each breed (Fig 5; S1 Fig). In both cases, phylogeny does not appear to significantly impact *AMY2B* copy number across breeds. Instead, our results indicate that starch intake appears to have influenced *AMY2B* copy number. For example, although both phylogenies reveal that the Shar Pei is more closely related to the Alaskan Malamute, Siberian Husky, and Japanese dogs [12] (Fig 5), the Shar Pei has a higher average copy number and a lower range in possible CNVs more similar to that of the Pekingese.

### Wolf introgression does not explain the low *AMY2B* CNV in low-starch breeds

**Fig 6:**
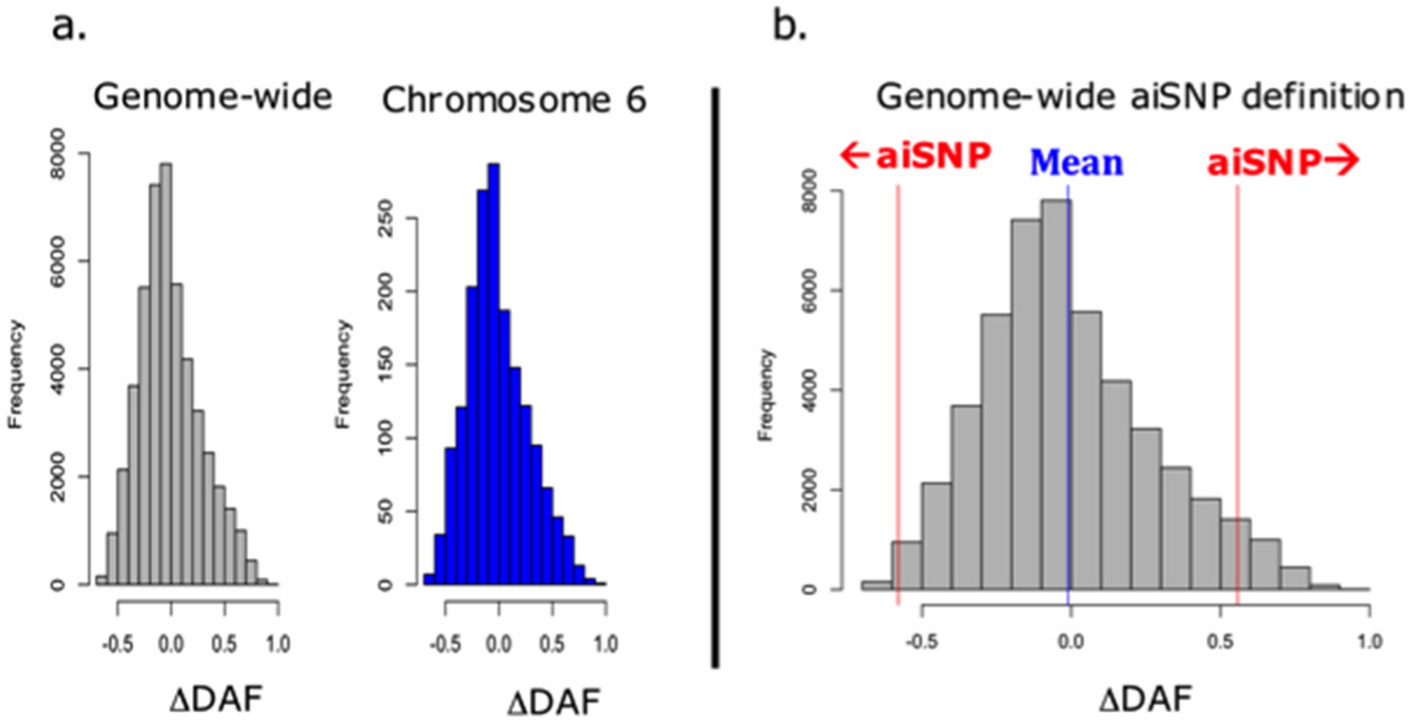
Distribution of dog-wolf difference of derived allele frequency (DAF). (**a**) There is no significant difference between the distribution of DAF genome-wide on chromosome 6 (Welch Two Sample t-test, p-value = 0.5867.) (**b**) Ancestry Informative SNPs (aiSNPs) are defined as those with an absolute DAF greater than two standard deviations from the genome-wide mean. Threshold are greater than 0.557 or less than -0.579.

Finally, to investigate whether introgression from wolves explains the *AMY2B* low copy number in the low-starch breeds, we determined the difference of derived allele frequency (ΔDAF) between our dog samples and our wolf samples for every locus in the genome for which we had genotype data (Methods). As a control, we confirmed that there was no difference between the genome-wide ΔDAF distribution and that on chromosome 6 (Fig 6) (Welch Two Sample t-test, p-value = 0.5867). We then defined “ancestry informative SNPs” (hereafter, “aiSNPs’”) as those with a ΔDAF with an absolute value greater than two standard deviations higher than the genome-wide mean (see Fig 6).

If the high frequency of low *AMY2B* copy numbers in the Alaskan Malamute, Siberian Husky, and the Japanese dogs is due to introgression from wolves, we would expect that at aiSNP loci within the *AMY2B* genomic region, low-starch breeds would have the “wolf allele” (the allele that is at high frequency in wolves) at higher frequency than the high-starch breeds. We identified three aiSNPs in the *AMY2B* region of the genome. Of the two dog breeds with the highest frequency of the “wolf” allele at these three loci, one was a high-starch, high-CNV breed, and the other was a low-starch, low-CNV breed (S2 Table). Therefore, these data indicate that it is unlikely that wolf introgression explains the low copy number of the low starch-breeds at this locus. For consistency, we repeated this procedure on the *GCKR* and *PHYH* loci and similarly found no correlation between ΔDAF at aiSNPs and starch intake (S3 and S4 Tables).

## Discussion

In this study, we investigated the relationship between diet and CNVs in three metabolic genes in the context of the evolutionary forces that have potentially operated since initial dog domestication. Of these three loci, only copy number variation for *AMY2B*, a gene with important roles in starch digestion [15,40], correlated with diet. While it is possible that *GCKR* or *PHYH* copy number were influenced by positive selection, we did not find evidence that either dietary intake of sugar or phytanic acid drove positive selection at these loci, respectively.

Specifically, our findings indicate that it is unlikely that sugar intake drove positive selection in *GCKR* CNV. We find no evidence that positive selection acted on this locus, though this study cannot definitively rule out the possibility of selection. Results from our aCGH analysis indicate that unusual pressures may have acted on *GCKR* CNV (S2 Fig) and we cannot exclude the possibility that other factors drove positive or negative selection at this locus, or that CNV at this locus was predominately influenced by random forces. Although it is primarily expressed in the liver, *GCKR* is also expressed in the ovaries and the adrenal glands of humans [41]. In dogs, *GCKR* expression has been identified in colonic mucosa [42]. It is possible that the expression profile of humans is conserved in dogs, or that *GCKR* plays a key unique role in the colonic mucosa of dogs, and that selection on *GCKR* CNV provides functional benefits other than maintenance of blood glucose homeostasis. Additionally, while our sample sizes are limited, it is unlikely that *GCKR* CNV alone plays a role in diabetes risk in dogs. Further research is needed to determine whether specific *GCKR* haplotypes are associated with increased or decreased risk of diabetes in dogs.

Our copy number analysis and results obtained for *PHYH* were also inconclusive. We identified that CNV at *PHYH* were variable with high CNV values being quite inaccurate and difficult to determine. When the available ddPCR data were analyzed on the aCGH distribution, the *PHYH* coordinate fell well outside of the cluster of copy number variable loci, indicating that no other surveyed loci had a profile similar to that of the *PHYH* ddPCR results and reflecting error in copy number calls. This inaccuracy reflects that, with all CNV computation methods used to date, the detection and resolution of high-copy number genotypes assumes high error (Methods). This highlights a broader need for technologies with increased accuracy in copy number resolutions especially for high copy variants. Without reliable copy number reads especially for those at the extreme high end of the distribution (and likely reflecting natural selection), it is difficult to speculate what selective factors may have driven copy number changes within each dog breed. However, given that dietary phytanic acid is toxic at high concentrations [43,44] and that it is an activator of genetic pathways involved in non-shivering thermogenesis [45], further analysis of *PHYH* function and CNV detection, using better methods when they become available, should examine if copy number balances the relative importance of each pathway.

Importantly, data obtained from ddPCR and aCGH suggested that *AMY2B* copy number may have been subject to positive selection in multiple breeds that consumed starch-rich diets. Because aCGH data were only available for the Alaskan Malamute and the Shar Pei, the null hypothesis cannot be fully tested in other breeds. However, the pattern of *AMY2B* CNV observed in all dogs that consumed high starch is similar, as is the pattern observed in all dogs that consumed low starch. Analysis of global trends in *AMY2B* CNV in a variety of breeds also showed that this pattern is not isolated to the six breeds examined in-depth. Although structural instability in this genomic region could create a high degree of variability in copy number variation, there is no evidence that the *AMY2B* region shows an unusually high level of instability [46] and any instability would be expected to affect all dog breeds randomly, and therefore does not explain our finding of the correlation between CNV and starch consumption.

These findings also extend species-wide. For example, when absolute latitude is used as a proxy for starch intake, global variation in *AMY2B* copy number mirrors results obtained from ddPCR. Indeed, *AMY2B* variation increases with increasing latitude, and although latitude does not predict copy number, as starch consumption decreases, variation in copy number increases.

These findings expand upon the recent study of Axelsson and colleagues [15], which found that *AMY2B* copy number is substantially increased in domestic dogs relative to wolves. This study presents evidence that in dog breeds that were exposed to starch-rich diets, positive selection continued to influence *AMY2B* copy number after this initial copy number expansion. Our findings also indicate that in dog breeds that adopted starch poor diets after copy number expansion, copy number likely evolved via genetic drift or via weak negative selection.

Previously, it has been reported that the Siberian Husky has not experienced copy number expansion at the *AMY2B* locus, suggesting that “the *AMY2B* copy number expansion was not fixed across all dogs early in the domestication process” [7]. This was inferred because in those huskies sampled *AMY2B* CNV ranged from three to four. On the contrary, with our expanded sampling strategy and use of ddPCR, which has slightly better ability to accurately call high CNV genotypes, we observed a copy number range from 2–15 in huskies, which, like all other breeds that have been surveyed, suggests that the copy number expansion event may likely have occurred prior to the split of modern major breeds worldwide. Of the 17 huskies we included in this study, four had a CNV of four or lower, which likely reflects genetic drift rather than possible introgression of alleles from wolves, though we cannot rule out introgression by other low CNV breeds. In summary, our observed patterns further validate that dogs experienced copy number expansion and that variation in CNV distribution likely reflects early positive selection at the *AMY2B* locus after initial expansion [15] and the possible impact of genetic drift or weak negative selection on the locus thereafter.

Importantly, the Japanese dog breeds we sampled may represent breeds in transition between drift and positive selection. It is likely that both breeds lived among the ancient hunter-gatherer cultures that existed prior to the agriculturalists that began dominating Japan roughly 2000 years ago [47]. The timing of this transition for each breed is not known, and may have differed for individual dogs. Although Japanese dogs display decreased mean CNV and increased variation compared to the Pekingese or Shar Pei, they have higher mean CNV and less variation than the Alaskan Malamute or Siberian Husky. This likely does not reflect the effects of island biogeography given that their variation is higher than nearby continental breeds, but could reflect a transition between a low- and high-starch diet that the breeds experienced. Because the transition to consuming a high-starch diet was likely recent in these breeds histories, and because of limited sampling, the Japanese breeds were included in the low-starch diet category.

These findings provide support for the idea that starch-rich diets were consumed after *AMY2B* CNV expansion, and likely before global migration of the ancestors to modern dogs. Given the genetic patterns identified in this study, we propose a model in which starch-rich diets predominated among most dog breeds coinciding with or shortly after the *AMY2B* CNV expansion and proliferation.

Although our findings suggest that *AMY2B* copy number expansion was likely influenced by positive selection pressure due to high-starch intake, we still find several individuals in low-starch breeds with high *AMY2B* copy numbers. For example, of Alaskan Malamutes sampled, close to 1/3 of individuals have a high CNV. We see three possible scenarios that could explain this finding. Scenario one calls for relatively recent positive selection for the high CNV, such that this pressure leads to the high CNV in already-differentiated high-starch dog breeds. In this case, the presence of some high copy numbers in the low-starch breeds is due to recent admixture between high- and low-starch dog breeds. We consider this scenario to be unlikely as there is no evidence of such introgression [48]. Both scenarios two and three place positive selection for the high CNV earlier in dog domestication, before the high- and low-starch breeds differentiated from each other. In scenario two, the low copy numbers in low-starch dogs is due to admixture between these breeds and wolves, which brought in low CNV haplotypes into the low-starch population. Given that our study found no evidence that wolf introgression is related to *AMY2B* copy number, we reject this scenario.

We therefore find a third scenario to be the most likely. We propose a model in which starch-rich diets predominated among most dogs after *AMY2B* expansion and before migration and differentiation of high- and low-starch breeds, coinciding with local starch consumption by humans. In turn, high starch consumption created positive selective pressure that likely acted on standing variation in *AMY2B* CNV to increase copy number species-wide. As some dog breeds migrated away, the selection pressure for high *AMY2B* CNV was maintained if dogs continued to consume starch-rich diets, or *AMY2B* CNV was subject to genetic drift if starch-poor diets were adopted. In the case of persistent positive selection, variation in *AMY2B* was kept low as selection pushed copy numbers toward an adaptive optimum. In the case of relaxed selection and genetic drift, variation increased throughout the dog breed and in certain locations, variation increased throughout the dog breed, possibly combined with positive selection on the low copy number variant in these populations.

In order to further test our hypotheses, future research should analyze *AMY2B* haplotype diversity using structural analyses in a variety of dog breeds in order to assess age and global distribution of haplotypes as has been carried out for the *AMY2A* locus in humans using 1000 Genomes datasets [49]. Future analyses would also be aided by improved copy number detection technologies. Particularly, it will be important to discern haploid copy number in order to detect selection via traditional methods. Finally, at this time little is understood regarding the functional ramifications of increased copy number and so future experiments should be targeted to these goals.

## Materials and Methods

### Dog samples

To investigate the relationship between diet and genetic polymorphisms located in and around key metabolic genes, DNA samples and dietary information were collected from multiple dog breeds (S5–S8 Tables). Sixty-two buccal swab samples were taken from six different dog breeds. These breeds are more genetically distinct than breeds created in the last few hundred years [12,39]. Dietary information was also collected from the Human Relations Area Files (OCM code 220) and from archaeological and isotopic analyses [5,18,50] and in general, dogs had variable dietary intake during their breed histories. CNV was surveyed in the genes *AMY2B*, *GCKR*, and *PHYH*.

For statistical analysis of *AMY2B*, CNV calls were also aggregated from other published studies [7,15,22]. Eighteen additional CNV calls from the same six breeds were used, bringing the sample total to eighty. For regression analyses, 172 additional *AMY2B* CNV calls from sixty-five other dog breeds were also included [7,15,22]. For these tests only, 252 total samples were used from seventy-one distinct breeds.

Finally, array-based comparative genomic hybridization (aCGH) data were obtained for four Alaskan Malamutes and five Shar Peis [34]. No *AMY2B* CNV calls were made on these samples; however, this data provided a comparative framework for analyses of other *AMY2B* CNV calls.

### DNA sample collection and processing

DNA samples were swabbed from the buccal side of each dog’s cheek using VWR Foam-Tipped Swab (catalog # 89009–338). Age and sex were not regarded, except all dogs sampled were older than six months of age. Following standard procedures, each sample was placed in an open 2.0 mL tube to dry for two hours and then closed and stored at -20°C until DNA extraction. DNA was extracted with the QIAamp mini DNA kit (Qiagen) using the Buccal Swab Protocol (Qiagen). Extracted samples were stored in 150 μL Buffer AE (Qiagen) at -20°C.

All dog samples were collected with owner’s permission. We received exemption from the oversight of the Institutional Animal Care and Use Committee of Harvard University. No dogs were hurt or adversely affected by the collection process.

### Copy number variation assays

Three CNV assays were performed on all collected samples. All copy number variants were determined using droplet digital PCR (ddPCR). Previously, real-time PCR (qPCR) has been used to study CNV in select breeds [7,15], however qPCR has limited ability to discern high copy number gene duplications [51,52], ddPCR allows absolute measure of oligonucleotides that are separated into an emulsified oil solution. This method generates a more accurate estimation of DNA copy number by decreasing the potential exponential error in a reaction and by producing technical error estimates for a single replicate. ddPCR was performed using QX200 Droplet Digital PCR system designed by Bio-Rad. ddPCR assay for *AMY2B* was performed as described in Arendt et al. [22]. ddPCR assays for *GCKR* and *PHYH* were adapted from Nicholas et al. [33]. For all assays, thermocyler settings were programmed as described by manufacturer (Bio-Rad) and under the following conditions: Amplification was done under the following conditions: one cycle at 95°C for 10 minutes, 40 cycles at 94°C for 30 seconds and 60°C for 1 minute, and one cycle at 98°C for 10 minutes. DNA was digested by restriction enzyme DraI (New England Bio Labs), allowing for separation of individual gene copies for better segregation and quantification in each droplet. ddPCR settings were sufficient to inactivate the enzyme. Restriction digests were diluted at varying concentrations from 10X to 1X and tested for replication. DNA concentrations varied after extraction, however because ddPCR does not have strict parameters for input DNA concentration, equal volumes of DNA were added to the assays regardless of concentration. Concentration of the added 2 μL of DNA ranged from .055-31.6 ng/μL. After ddPCR assays were completed, copy number was regressed by DNA concentration to see if a relationship was produced between concentration and copy number. No correlation was identified. Because of the nature of ddPCR, technical error reports replaced traditional replicates (S6–S8 Tables). Raw copy number data were rounded to the nearest whole number and with technical error estimates (S6–S8 Tables). Primer and probe sequences for *AMY2B*, *GCKR*, *PHYH* and reference genes are located in S9 Table.

### Statistical analyses

Statistical analyses of copy number variants were performed using the StatPlus package for Microsoft Excel, as well as in R. Modeling and graphics were created using Excel and R. The Robust Rank Order Test was employed to test differences in medians because it does not assume that the populations have the same shape or variances [53]. Fligner-Killeen test was employed to test homogeneity of variances of copy number in dogs that consumed different diets [54].

### aCGH analysis

To determine if *AMY2B* CNV mean log_2_ ratio deviated substantially from the null distribution of other copy number variable sites in the genome, we analyzed residuals of *AMY2B* to a linear regression line fit to the aCGH data. 156 CNV sites were present in at least one Alaskan Malamute and one Shar Pei in the aCGH data. aCGH data are presented as log_2_ ratios that represent the amount of genetic material present relative to a common control (CanFam2) at each surveyed SNP. The mean log_2_ ratio was calculated for each site in both the Alaskan Malamute and the Shar Pei by averaging all log_2_ ratios within each copy number variable site. Next, mean log_2_ ratios for the Alaskan Malamute and the Shar Pei were plotted against each other to produce a null distribution for the relationship between standard genomic CNV in the Alaskan Malamute and the Shar Pei.

The mean log_2_ ratio for *AMY2B* in Alaskan Malamutes and Shar Peis were added to the null distribution. Mean log_2_ ratios were calculated relative to CanFam2 and by using the *AMY2B* CNV data produced by ddPCR.

A linear regression analysis was conducted on the distribution of Alaskan Malamute and Shar Pei mean log_2_ ratios to determine if *AMY2B* CNV deviated (Fig 3). If no difference in variation existed between synonymous copy number variable regions, then the breed log_2_ ratios would be similar, even if dissimilar from zero. Any copy number variant with a mean log_2_ ratio that deviates substantially from the null distribution may have been subject to unusual evolutionary pressure in one or both of the breeds.

To determine how *AMY2B* compared to this null distribution, residuals were calculated from the linear regression model. Mean and standard deviation for these residuals were calculated and used to compare deviation in *AMY2B* CNV in these breeds. Because of limited samples size, this analysis was performed again taking into consideration other breeds from previously published studies employing qPCR datasets in order to generate a more robust null distribution (S2 Appendix).

### Regression of absolute latitude by CNV

Latitude at location of dog origin was used to determine breed-level designation of starch intake for breeds with published *AMY2B* CNV calls. Latitude was next adjusted to reflect absolute distance from the equator. Absolute latitude was next regressed by *AMY2B* CNV of 252 dogs to determine if starch intake predicted copy number, and calls were separated into two categories: those below 40° latitude (n = 75) and those above 40° latitude (n = 177). A Fligner-Killeen test was run between the two groups to test homogeneity of variances [54].

### Difference of derived allele frequency and introgression analyses

The difference of derived allele frequency (ΔDAF) was determined according to the following formula, *ΔDAF = DAF*_*dogs*_*—DAF*_*wolves*._ Genotype information came from analyses conducted by vonHoldt and colleagues [12]. The mean and standard deviation of ΔDAF throughout the genome was determined using the R “mean()” and “sd()” functions. Ancestry informative SNPs (aiSNPs) were those with a ΔDAF with an absolute value greater than two standard deviations above the mean. In determining the region in which to search for aiSNPs for each gene, our goal was to insure that we included any potentially relevant aiSNPs in either the coding or non-coding regions of each gene. We therefore initially considered the region between the transcription start sites of the flanking genes on either side of our genes of interest. Given the relatively poor annotation of the dog genome, we additionally further expanded these regions by 500kb on either side due to a convention established by vonHoldt et al. [12] in which wolf-introgressed haplotypes in dogs were found to be 500kb in length. The coordinates we considered for each gene and the location of the aiSNPs are shown in S10 Table.

## Supporting Information

S1 Fig Alternate cladogram of dog relations. Cladogram depicting the relationship between the dog breeds evaluated in this study and diploid AMY2B copy number.
(PDF)

S2 Fig Additional results from analysis of aCGH data. Relationship between Shar Pei and Alaskan malamute mean log2 ratios for 157 copy number variable sites in the dog genome. *GCKR* deviates from the null distribution. The location of *PHYH* likely reflects error in ddPCR measurements due to high copy number.
(PDF)

S1 Appendix *AMY2B* CNV with supplemental data. Analyses of *AMY2B* CNV using additional datasets.
(PDF)

S2 Appendix Array-Based Comparative Genomic Hybridization Analysis for Multiple Breeds. Analyses of aCGH datasets across a number of dog breeds.
(PDF)

S1 Table Changes in *GCKR* copy number relative to reference sequence CanFam2, and diabetes odds risk for specific breeds. (PDF)

S2 Table Frequency of derived allele in *AMYB2* ancestry informative SNPs for high and low starch dog breeds. (PDF)

S3 Table Frequency of derived allele in *GKCR* ancestry informative SNPs in high and low starch dog breeds. (PDF)

S4 Table Frequency of derived allele in *PHYH* ancestry informative SNPs in high and low starch dog breeds. (PDF)

S5 Table Traditional dietary intake of specific dog breeds. (PDF)

S6 Table Diploid *AMY2B* copy number and technical error estimates from ddPCR. (PDF)

S7 Table Diploid *GCKR* copy number and technical error estimates from ddPCR. (PDF)

S8 Table Diploid *PHYH* copy number and technical error estimates from ddPCR. (PDF)

S9 Table Primer and Probe sequences used for ddPCR assays for target and reference genes. (PDF)

S10 Table Coordinates of genes and location of aiSNPs used for calculation of ΔDAF and introgression analysis. (PDF)
