# Supplementary material for: Dietary Variation and Evolution of Gene Copy Number among Dog Breeds
Source: PLoS One. 2016 Feb 10;11(2):e0148899. doi: 10.1371/journal.pone.0148899 (PMC4749313; doi:10.1371/journal.pone.0148899)
Supplement: S1 Fig — Cladogram depicting the relationship between the dog breeds evaluated in this study and diploid AMY2B copy number. (PDF) [file pone.0148899.s001.pdf]

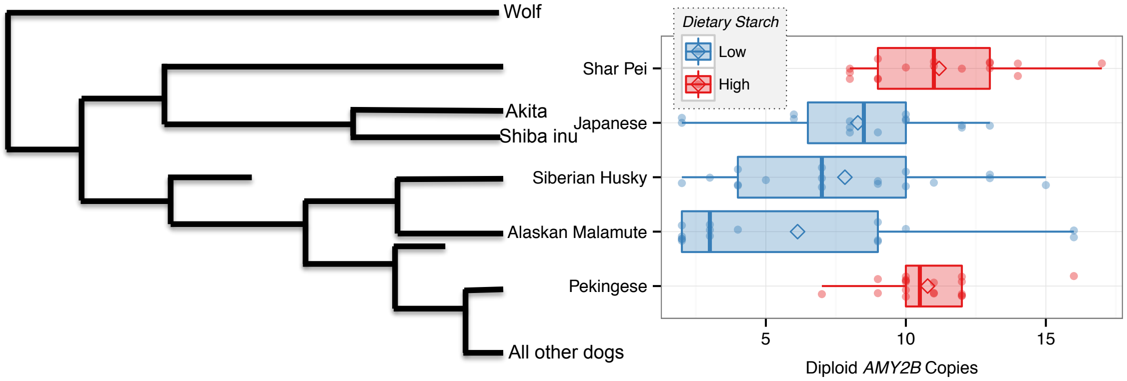

Figure S1. Cladogram depicting the relationship between the dog breeds evaluated in this study and diploid *AMY2B* copy number. Cladogram relations were determined from molecular marker data and was adapted from Parker et al., 2004.

1. Parker HG, Kim LV, Sutter NB, Carlson S, Lorentzen TD, Malek TB, et al. Genetic structure of the purebred domestic dog. Science (New York, NY). 2004;304(5674):1160-4.
