# Supplementary material for: Dietary Variation and Evolution of Gene Copy Number among Dog Breeds
Source: PLoS One. 2016 Feb 10;11(2):e0148899. doi: 10.1371/journal.pone.0148899 (PMC4749313; doi:10.1371/journal.pone.0148899)
Supplement: S2 Fig — Relationship between Shar Pei and Alaskan malamute mean log2 ratios for 157 copy number variable sites in the dog genome. GCKR deviates from the null distribution. The location of PHYH likely reflects error in ddPCR measurements due to high copy number. (PDF) [file pone.0148899.s002.pdf]

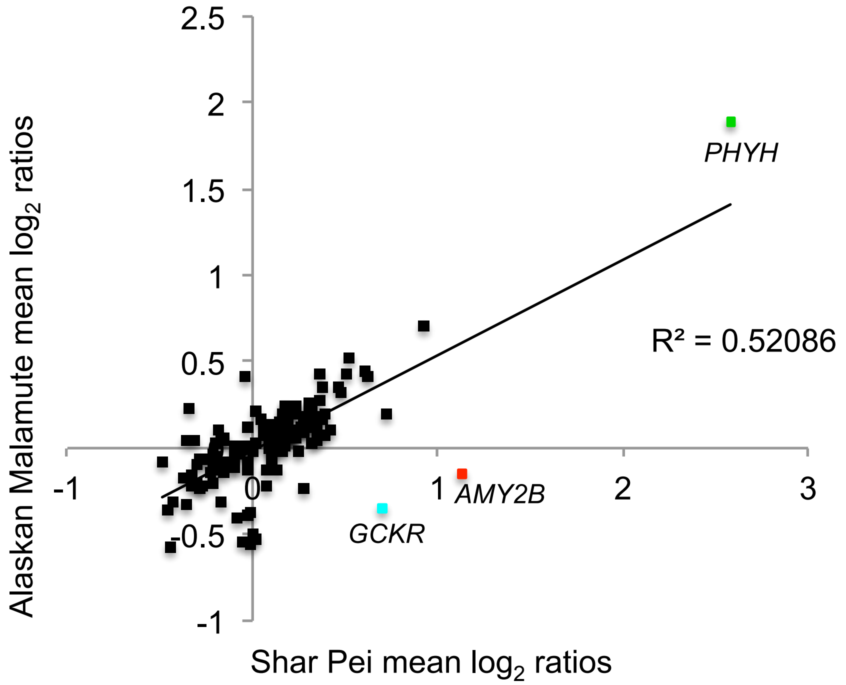

Figure S2. Additional results from analysis of aCGH data. Relationship between Shar Pei and Alaskan malamute mean log_2 ratios for 157 copy number variable sites in the dog genome. *GCKR* deviates from the null distribution. The location of *PHYH* likely reflects error in ddPCR measurements due to high copy number.
