# Supplementary material for: Dietary Variation and Evolution of Gene Copy Number among Dog Breeds
Source: PLoS One. 2016 Feb 10;11(2):e0148899. doi: 10.1371/journal.pone.0148899 (PMC4749313; doi:10.1371/journal.pone.0148899)
Supplement: S1 Appendix — Analyses of AMY2B CNV using additional datasets. (PDF) [file pone.0148899.s003.pdf]

Appendix S1: ***AMY2B* CNV with supplemental data**

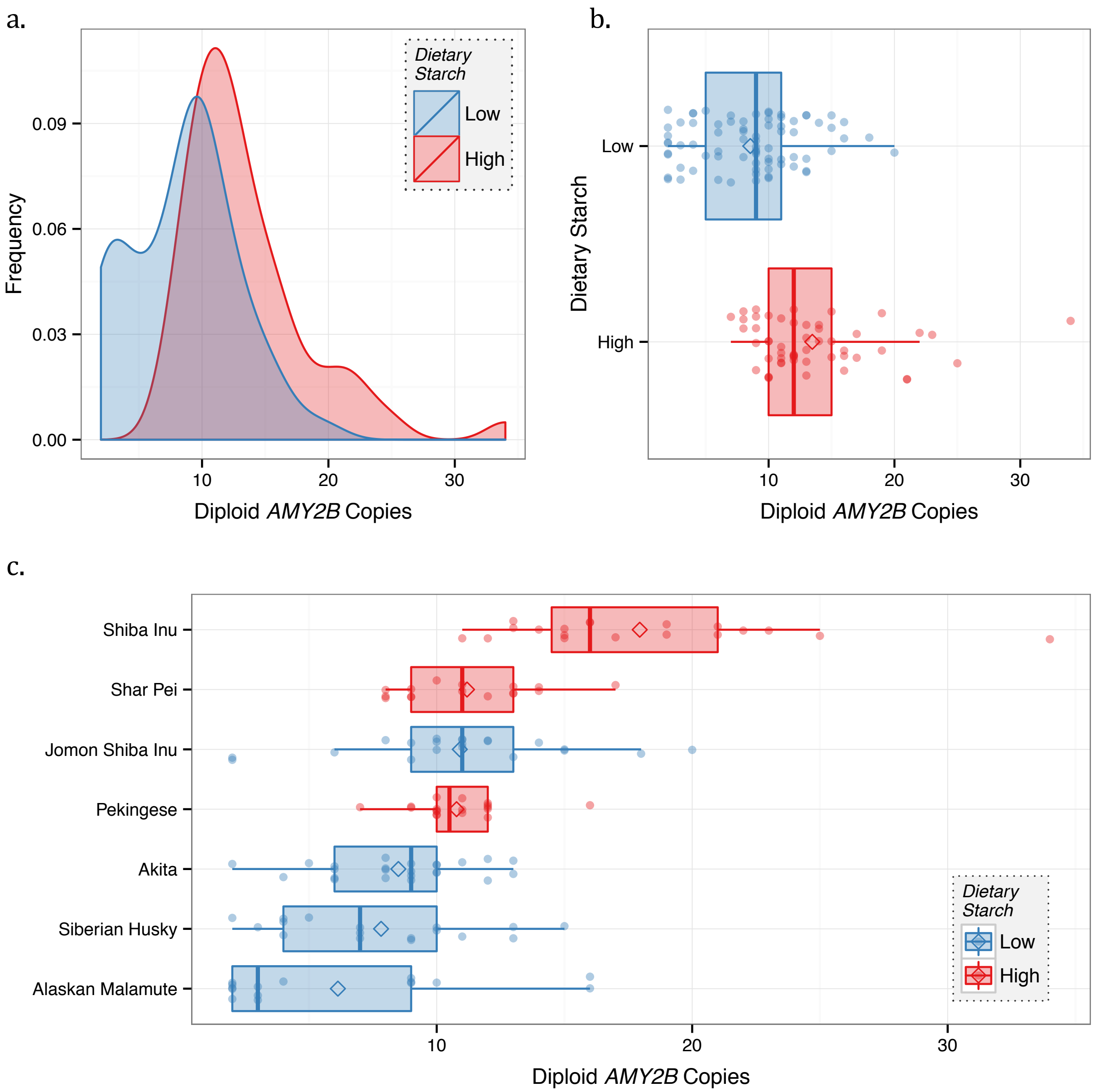

Figure 1: Diet and *AMY2B* copy number variation, including data from the recent Tonoike et al. (2015) (1) analysis of *AMY2B* copy number variation in dog breeds of Japanese descent. Because the authors used qPCR to determine copy number, and because some Shiba Inu samples had high copy number, these samples were excluded from the main text because of the error introduced by qPCR at high copy number. However, these data highlight that past selection may have shaped these differences, and that *AMY2B* copy number is not reliant on phylogeny. **a)** Density plot of ddPCR diploid *AMY2B* copy number for dogs that traditionally consumed high-starch diets and low-starch diets. Density reflects frequency with which a given diploid copy number appears in each population. **b)** Tukey boxplot of diploid *AMY2B* copy number for dogs that traditionally consumed high-starch diets and low-starch diets. **c)** Tukey boxplot of diploid *AMY2B* copy number for specific dog breeds that traditionally consumed high-starch diets and low-starch diets.

1. Tonoike A, Hori Y, Inoue-Murayama M, Konno A, Fujita K, Miyado M, et al. Copy number variations in the amylase gene (AMY2B) in Japanese native dog breeds. Animal genetics. 2015;46(5):580-3.
