# Supplementary material for: Dietary Variation and Evolution of Gene Copy Number among Dog Breeds
Source: PLoS One. 2016 Feb 10;11(2):e0148899. doi: 10.1371/journal.pone.0148899 (PMC4749313; doi:10.1371/journal.pone.0148899)
Supplement: S2 Appendix — Analyses of aCGH datasets across a number of dog breeds. (PDF) [file pone.0148899.s004.pdf]

Appendix S2: Array-Based Comparative Genomic Hybridization Analysis for Multiple Breeds.

aCGH data were used to determine whether unusual selective pressures or stochastic gene processes affected copy number variation in high- vs. low-starch breeds. Because aCGH information was only available for 9 high- or low-starch dogs (4 Alaskan Malamutes, 5 Shar Peis), these data were supplemented by 55 samples from 10 diverse dog breeds (Border Collie, Beagle, Brittany Spaniel, Boxer, Dachshund, Greyhound, German Shepard, Jack Russell Terrier, Labrador Retriever, and Standard Poodle) (1). aCGH data are presented as $\log_2$ ratios that represent amount of genetic material present relative to a common control (CanFam2) at each surveyed SNP. Within the 64 samples dogs, 350 loci were copy number variable in more than one individual and in at least two breeds. The mean $\log_2$ ratio was calculated for each site in each dog and averaged for each breed. The values for each breed were plotted against the values for the Alaskan Malamute and the Shar Pei, respectively (**Figure 1**). A linear regression was performed for each plot and residuals were calculated from each regression. These residuals were plotted on a histogram to create a null distribution of copy number among the dogs sampled. The mean $\log_2$ ratio for *AMY2B* in Alaskan Malamutes and Shar Peis were added to the null distribution. Mean $\log_2$ ratios were calculated relative to CanFam2 and by using the *AMY2B* CNV data produced by ddPCR (**Figure 2**). *AMY2B* remained a clear outlier to the null distribution. This indicates that it is likely that unusually selective pressures or stochastic forces influenced *AMY2B* copy number in either the Alaskan Malamute or the Shar Pei, or both.

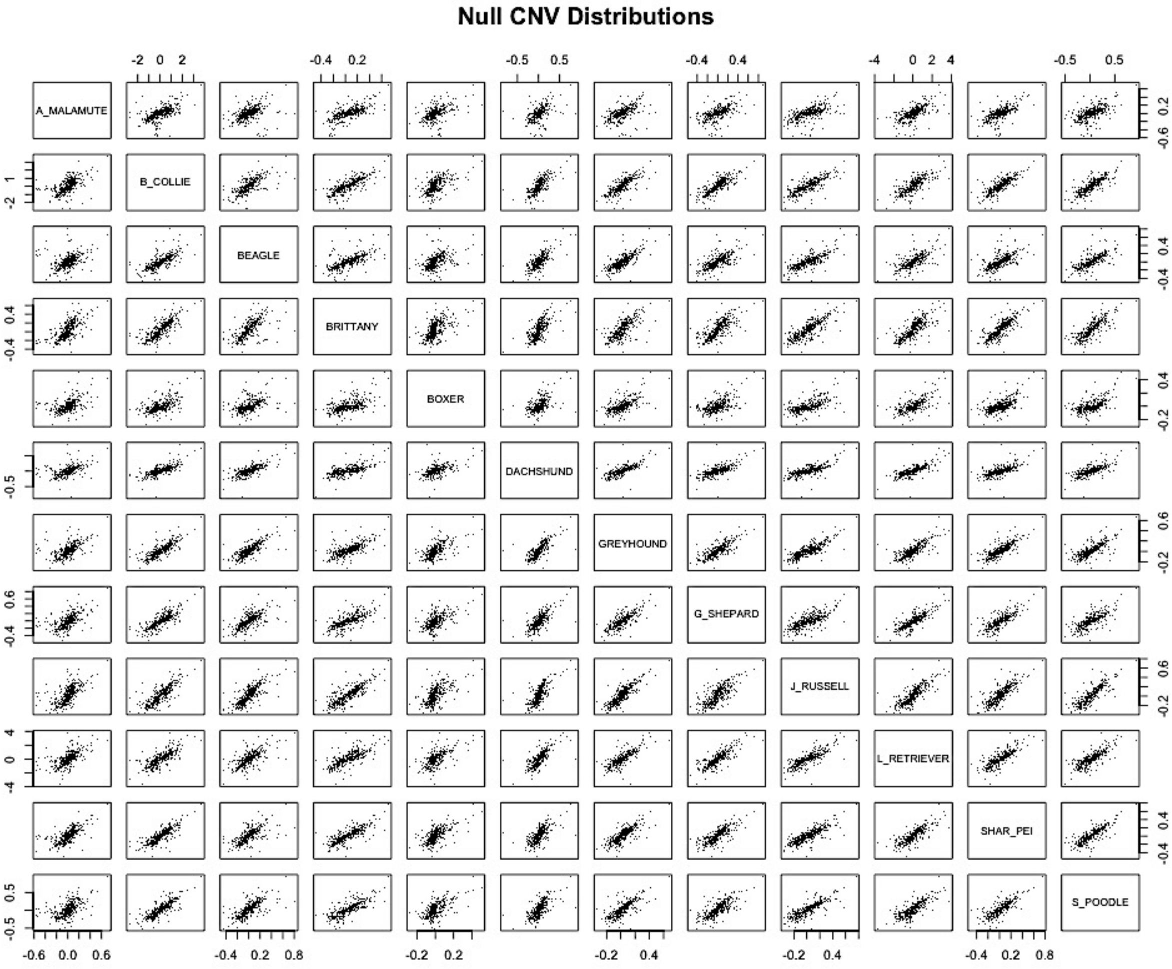

Figure 1: Scatter plot matrix of mean log_2 ratios from aCGH data.

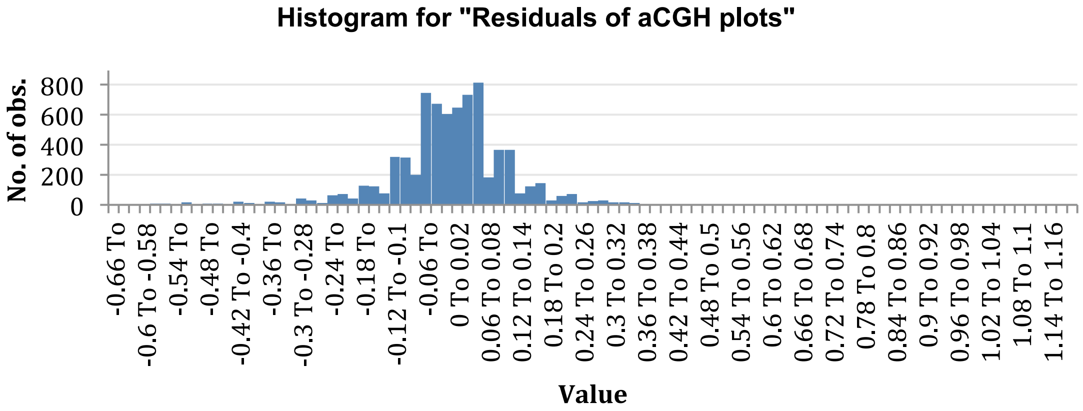

Figure 2: Histogram of residuals from each linear regression plotted against either the Alaskan Malamute or Shar Pei. *AMY2B* falls significantly outside of the histogram. 7371 observations are represented on the histogram, and *AMY2B* is 15.8 standard deviations away from the mean.

1. Nicholas TJ, Baker C, Eichler EE, Akey JM. A high-resolution integrated map of copy number polymorphisms within and between breeds of the modern domesticated dog. BMC genomics. 2011;12:414 (GEO http://www.ncbi.nlm.nih.gov/geo/ accession number GSE26170).
