# Supplementary material for: Dietary Variation and Evolution of Gene Copy Number among Dog Breeds
Source: PLoS One. 2016 Feb 10;11(2):e0148899. doi: 10.1371/journal.pone.0148899 (PMC4749313; doi:10.1371/journal.pone.0148899)
Supplement: S1 Table — (PDF) [file pone.0148899.s005.pdf]

Table S1. Changes in *GCKR* copy number relative to the reference sequence CanFam2, and diabetes odds risk for specific breeds.

Breed	# Gain	# Same	# Loss	Diabetes Odds Risk
Pekingese	12	1	2	.18[a]
Shar Pei	8	0	0	n/a[b]
Akita	7	0	0	n/a[b]
Shiba Inu	4	0	0	n/a[b]
Siberian Husky	14	0	1	1.53[c]; 3.46[d]
Alaskan Malamute	5	1	7	No increased risk[e]

Number of dogs in each breed with a gain, the same, or a loss of copy number in *GCKR* compared to the reference sequence CanFam2. [a, c, e](1), [b]no available data on diabetes odds risk, [d](2)

1. Guptill L, Glickman L, Glickman N. Time trends and risk factors for diabetes mellitus in dogs: analysis of veterinary medical data base records (1970-1999). Veterinary journal (London, England : 1997). 2003;165(3):240-7.

2. Catchpole B, Adams JP, Holder AL, Short AD, Ollier WE, Kennedy LJ. Genetics of canine diabetes mellitus: are the diabetes susceptibility genes identified in humans involved in breed susceptibility to diabetes mellitus in dogs? Veterinary journal (London, England : 1997). 2013;195(2):139-47.
