# Supplementary material for: Dietary Variation and Evolution of Gene Copy Number among Dog Breeds
Source: PLoS One. 2016 Feb 10;11(2):e0148899. doi: 10.1371/journal.pone.0148899 (PMC4749313; doi:10.1371/journal.pone.0148899)
Supplement: S2 Table — (PDF) [file pone.0148899.s006.pdf]

Table S2. Frequency of Derived Allele in *AMYB2* Ancestry Informative SNPs for High and Low Starch Dog Breeds

| aiSNP | Breed | Starch | Dfreq |
|---|---|---|---|
| **chr6.50074785** | Pekingese | High | 1 |
| | Saluki | High | 1 |
| | SharPei | High | 0.29 |
| | ShihTzu | High | 1 |
| | Akita | Low | 1 |
| | AlaskanMalamute | Low | 0.59 |
| | American Eskimo Dog | Low | 1 |
| | SiberianHusky | Low | 0.29 |
| **chr6.50081582** | Pekingese | High | 1 |
| | Saluki | High | 1 |
| | SharPei | High | 0.29 |
| | ShihTzu | High | 1 |
| | Akita | Low | 1 |
| | AlaskanMalamute | Low | 0.59 |
| | American Eskimo Dog | Low | 1 |
| | SiberianHusky | Low | 0.29 |
| **chr6.50089070** | Pekingese | High | 1 |
| | Saluki | High | 1 |
| | SharPei | High | 0.29 |
| | Shihtzu | High | 1 |
| | Akita | Low | 1 |
| | AlaskanMalamute | Low | 0.59 |
| | American Eskimo Dog | Low | 1 |
| | SiberianHusky | Low | 0.29 |

Column headings: aiSNP-location of the ancestry informative snp; Breed- name of dog breed; Starch- High or low starch consumption; Dfreq- derived allele frequency.
