# Supplementary material for: Dietary Variation and Evolution of Gene Copy Number among Dog Breeds
Source: PLoS One. 2016 Feb 10;11(2):e0148899. doi: 10.1371/journal.pone.0148899 (PMC4749313; doi:10.1371/journal.pone.0148899)
Supplement: S3 Table — (PDF) [file pone.0148899.s007.pdf]

Table S3. Frequency of Derived Allele in *GKCR* Ancestry Informative SNPs in High and Low Starch Dog Breeds

aiSNP	Breed	Starch	Dfreq
chr17.24309172	Pekingese	High	0.77
	Saluki	High	0.75
	Shar Pei	High	0.75
	Shih Tzu	High	0.90
	Akita	Low	1
	Alaskan Malamute	Low	1
	American Eskimo Dog	Low	0.50
	Siberian Husky	Low	0.14
chr17.24312880	Pekingese	High	0.77
	Saluki	High	0.59
	Shar Pei	High	0.70
	Shih Tzu	High	0.89
	Akita	Low	1
	Alaskan Malamute	Low	1
	American Eskimo Dog	Low	0.60
	Siberian Husky	Low	0.14
chr17.25295979	Pekingese	High	0.20
	Saluki	High	0.73
	Shar Pei	High	0.92
	Shih Tzu	High	0.33
	Akita	Low	0.95
	Alaskan Malamute	Low	0.14
	American Eskimo Dog	Low	0.93
	Siberian Husky	Low	0.32

Column headings: aiSNP-location of the ancestry informative snp; Breed- name of dog breed; Starch- High or low starch consumption; Dfreq- derived allele frequency.
