# Supplementary material for: Dietary Variation and Evolution of Gene Copy Number among Dog Breeds
Source: PLoS One. 2016 Feb 10;11(2):e0148899. doi: 10.1371/journal.pone.0148899 (PMC4749313; doi:10.1371/journal.pone.0148899)
Supplement: S4 Table — (PDF) [file pone.0148899.s008.pdf]

Table S4. Frequency of Derived Allele in *PHYH* Ancestry Informative SNPs in High and Low Starch Dog Breeds

aiSNP	Breed	Starch	Dfreq
chr2.25670153	Pekingese	High	0.36
	Saluki	High	0.38
	Shar Pei	High	0.58
	Shih Tzu	High	1
	Akita	Low	0.38
	Alaskan Malamute	Low	0.45
	American Eskimo Dog	Low	0.50
	Siberian Husky	Low	0.96

Column headings: aiSNP-location of the ancestry informative snp; Breed- name of dog breed; Starch- High or low starch consumption; Dfreq- derived allele frequency.
