# Supplementary material for: Dietary Variation and Evolution of Gene Copy Number among Dog Breeds
Source: PLoS One. 2016 Feb 10;11(2):e0148899. doi: 10.1371/journal.pone.0148899 (PMC4749313; doi:10.1371/journal.pone.0148899)
Supplement: S5 Table — (PDF) [file pone.0148899.s009.pdf]

Table S5. Traditional dietary intake of specific dog breeds.

Breed	Subsistence	Dietary Starch
Chinese Shar Pei	Horticulture/cereal agriculture	Pre agriculture: sago palms, banana, water chestnuts, lotus roots, arrowheads, ferns, Job's tears, acorns, sedge, bamboo some rice (1). Post horticulture/ agriculture: Rice, Chinese yam (2).
Pekingese	Gathering, cereal agriculture	Pre agriculture: acorn, foxtail millet, oak species, broomcorn, bean, roots, grasses, deer (3). Post agriculture: millet (4).
Akita and Shiba Inu	Hunter-gathering/cereal agriculture	Jõmon: limited bottle gourd, mung beans, millet, barley, burdock, rice, fish (5); Agriculturalists: rice cultivation (5); Ainu: seasonally available vegetables, fish, deer (6).
Siberian Husky	Hunter-gathering	Reindeer, freshwater fish (7, 8).
Alaskan Malamute	Hunter-gathering	Whale, seal, caribou, salmon, whitefish, saffron cod, northern pike, arctic grayling, burbot, and Pacific herring (7, 9).

References

1. Yang X, Barton HJ, Wan Z, Li Q, Ma Z, Li M, et al. Sago-Type Palms Were an Important Plant Food Prior to Rice in Southern Subtropical China. PloS one. 2013;8(5):e63148.
2. Weng Q. Human-environment interactions in agricultural land use in a South China's wetland region: A study on the Zhujiang Delta in the Holocene. GeoJournal. 2000;51(3):191-202.
3. Yang X, Yu J, Lü H, Cui T, Guo J, Ge Q. Starch grain analysis reveals function of grinding stone tools at Shangzhai site, Beijing. Sci China Ser D-Earth Sci. 2009;52(8):1164-71.
4. Pechenkina EA, Ambrose SH, Xiaolin M, Benfer Jr RA. Reconstructing northern Chinese Neolithic subsistence practices by isotopic analysis. Journal of Archaeological Science. 2005;32(8):1176-89.
5. Habu J. Ancient Jomon of Japan. Cambridge, UK: Cambridge University Press; 2004. 350 p.
6. Shinichiro T. The Ainu of northern Japan : a study in conquest and acculturation. Philadelphia: American Philosophical Society; 1960.
7. Porsild AE. Edible Plants of the Arctic. 1953. 1953;6(1).
8. Kozlov A, Vershubsky G, Kozlova M. Indigenous Peoples of Northern Russia: Anthropoly and Health. 2007.
9. Driver H. Indians of North America. 2 ed. Chicago: University of Chicago Press; 1969.
