# Supplementary material for: Dietary Variation and Evolution of Gene Copy Number among Dog Breeds
Source: PLoS One. 2016 Feb 10;11(2):e0148899. doi: 10.1371/journal.pone.0148899 (PMC4749313; doi:10.1371/journal.pone.0148899)
Supplement: S6 Table — (PDF) [file pone.0148899.s010.pdf]

Table S6: Diploid *AMY2B* copy number and technical error estimates from ddPCR

Breed	Sample	Diploid *AMY2B* CN Estimate	Diploid *AMY2B* CN	Poisson Max CN Estimate*	Poisson Min CN Estimate*
Alaskan malamute	AM-1	1.93	2	2.14	1.8
Alaskan malamute	AM-2	10.28	10	10.82	9.86
Alaskan malamute	AM-3	2.04	2	2.26	1.95
Alaskan malamute	AM-4	15.8	16	17.3	15.1
Alaskan malamute	AM-5	9.4	9	10	8.2
Alaskan malamute	AM-6	1.97	2	2.13	1.87
Alaskan malamute	AM-7	1.81	2	2.07	1.65
Alaskan malamute	AM-8	9	9	9.5	8.4
Alaskan malamute	AM-9	3.25	3	3.35	3
Alaskan malamute	AM-10	2.87	3	3.02	2.71
Alaskan malamute	AM-11	2.9	3	3.04	2.74
Alaskan malamute	AM-12	8.93	9	9.46	8.53
Alaskan malamute	AM-13	1.88	2	2.1	1.74
Shar Pei	CSP-1	11.8	12	11.4	8.6
Shar Pei	CSP-2	9.4	9	10.7	8.5
Shar Pei	CSP-3	13.6	14	15.5	10.2
Shar Pei	CSP-4	12.7	13	13.2	10.4
Shar Pei	CSP-5	9.3	9	11.8	9.7
Shar Pei	CSP-6	10.6	11	15.1	11
Shar Pei	CSP-7	8.6	9	9.9	7.2
Shar Pei	CSP-8	11.2	11	12.1	12.1
Japanese	AK-1	9.9	10	10.1	9.1
Japanese	SI-1	1.81	2	2.11	1.73
Japanese	SI-2	10	10	10.6	9.4
Japanese	SI-3	2	2	2.15	1.94
Japanese	SI-4	11.8	12	13	10.5
Japanese	AK-2	12.6	13	13.7	11.5
Japanese	AK-3	9.5	10	10.2	8.9
Japanese	AK-4	8.93	9	9.4	8.46
Japanese	AK-5	8.1	8	8.45	7.74
Japanese	AK-6	7.6	8	8.3	6.9
Japanese	AK-7	12.3	12	13	11.7
Pekingese	PK-1	10.1	10	12	8.6
Pekingese	PK-2	9.9	10	11.3	9.6
Pekingese	PK-3	9	9	11.1	8.4
Pekingese	PK-4	11	11	13.3	11.2
Pekingese	PK-5	12.1	12	13.9	10.3
Pekingese	PK-6	9.5	10	10.6	8.5
Pekingese	PK-7	10.1	10	11.4	9

Pekingese	PK-8	8.7	9	10	7.9
Pekingese	PK-9	12.1	12	12.1	10.4
Pekingese	PK-10	11.5	12	11.1	8.1
Pekingese	PK-11	10.9	11	11.9	10.7
Pekingese	PK-12	10.4	10	11.6	8.9
Pekingese	PK-13	7	7	8.8	5.4
Pekingese	PK-14	12.2	12	12.8	11.3
Pekingese	PK-15	10.9	11	11.6	9.7
Siberian husky	SH-1	4.1	4	4.18	3.76
Siberian husky	SH-2	15.5	15	15.4	14.1
Siberian husky	SH-3	9.13	9	9.18	8.54
Siberian husky	SH-4	4.12	4	4.13	3.83
Siberian husky	SH-5	9.57	10	9.81	8.96
Siberian husky	SH-6	10.6	11	10.99	10
Siberian husky	SH-7	13.2	13	13.9	12.7
Siberian husky	SH-8	6.83	7	7.09	6.56
Siberian husky	SH-9	12.9	13	14.1	12.4
Siberian husky	SH-10	8.9	9	9.4	8.2
Siberian husky	SH-11	9.89	10	10.4	9.44
Siberian husky	SH-12	7.15	7	7.3	6.45
Siberian husky	SH-13	5.37	5	5.43	5.01
Siberian husky	SH-14	2.02	2	2.1	1.94
Siberian husky	SH-15	4.18	4	4.38	4

*Droplet Digital PCR generates maximum and minimum technical error estimates from one replicate.
