# Supplementary material for: Dietary Variation and Evolution of Gene Copy Number among Dog Breeds
Source: PLoS One. 2016 Feb 10;11(2):e0148899. doi: 10.1371/journal.pone.0148899 (PMC4749313; doi:10.1371/journal.pone.0148899)
Supplement: S7 Table — (PDF) [file pone.0148899.s011.pdf]

Table S7: Diploid *GCKR* copy number and technical error estimates from ddPCR

Breed	Sample	Diploid *GCKR* CN Estimate	Diploid *GCKR* CN	Poisson Max CN Estimate*	Poisson Min CN Estimate*
Alaskan malamute	AM-1	6.01	6	7.8	5.4
Alaskan malamute	AM-2	3.65	4	8.3	6.9
Alaskan malamute	AM-3	3.49	3	10.9	8
Alaskan malamute	AM-4	4.50	5	10	8.7
Alaskan malamute	AM-5	3.80	4	7.6	5.5
Alaskan malamute	AM-6	4.29	4	11.6	9.1
Alaskan malamute	AM-7	4.40	4	8.1	6.3
Alaskan malamute	AM-8	4.05	4	5.2	4.1
Alaskan malamute	AM-9	3.34	3	13.5	6.5
Alaskan malamute	AM-10	7.90	8	3.9	2.2
Alaskan malamute	AM-11	6.27	6	6.32	5.58
Alaskan malamute	AM-12	6.20	6	11	7.5
Alaskan malamute	AM-13	6.90	7	11.1	6
Shar Pei	CSP-1	8.40	8	6.9	5.6
Shar Pei	CSP-2	10.70	11	4.7	3.4
Shar Pei	CSP-3	7.30	7	6.28	5.47
Shar Pei	CSP-4	11.30	11	6.49	5.53
Shar Pei	CSP-5	8.50	9	10.8	6.1
Shar Pei	CSP-6	15.00	15	13.4	8
Shar Pei	CSP-7	6.70	7	9.1	5.6
Shar Pei	CSP-8	12.60	13	14	8.6
Japanese	AK-1	5.88	6	9.9	7.2
Japanese	SI-1	5.90	6	20	10
Japanese	SI-2	6.00	6	7.9	5.6
Japanese	SI-3	9.50	10	14.1	11
Japanese	SI-4	5.40	5	7	4.8
Japanese	AK-2	5.90	6	6.8	5.7
Japanese	AK-3	5.56	6	10.2	8.9
Japanese	AK-4	9.20	9	6.3	4.5
Japanese	AK-5	8.60	9	3.99	3.3
Japanese	AK-6	6.50	7	3.8	3.18
Japanese	AK-7	7.00	7	5.2	3.9
Pekingese	PK-1	6.60	7	4.4	3.2
Pekingese	PK-2	7.60	8	4.62	3.96
Pekingese	PK-3	9.40	9	5.2	3.7
Pekingese	PK-4	9.40	9	4.41	3.69
Pekingese	PK-5	6.50	7	3.54	3.13
Pekingese	PK-6	10.30	10	14.5	12.6
Pekingese	PK-7	7.20	7	9.85	8.91

Pekingese	PK-8	4.70	5	8.35	7.46
Pekingese	PK-9	10.00	10	11	9.8
Pekingese	PK-10	3.00	3	12.1	10.5
Pekingese	PK-11	5.95	6	11.6	9.9
Pekingese	PK-12	9.30	9	12	10.4
Pekingese	PK-13	8.50	9	11.5	10.2
Pekingese	PK-14	6.30	6	23	6
Pekingese	PK-15	4.10	4	16.4	12.2
Siberian husky	SH-1	13.60	14	8.8	7.4
Siberian husky	SH-2	9.38	9	12.3	10.5
Siberian husky	SH-3	7.91	8	10.9	9.8
Siberian husky	SH-4	10.40	10	4.27	3.81
Siberian husky	SH-5	11.30	11	13.8	12.1
Siberian husky	SH-6	10.80	11	8.5	7.2
Siberian husky	SH-7	11.20	11	6.76	5.77
Siberian husky	SH-8	10.90	11	6.8	5.6
Siberian husky	SH-9	14.00	14	7.5	6.3
Siberian husky	SH-10	14.30	14	6.4	5.4
Siberian husky	SH-11	8.10	8	6.01	5.11
Siberian husky	SH-12	11.40	11	9.7	8.6
Siberian husky	SH-13	10.40	10	9.1	8
Siberian husky	SH-14	4.04	4	7	5.9
Siberian husky	SH-15	12.90	13	7.6	6.3

*Droplet Digital PCR generates maximum and minimum technical error estimates from one replicate.
