# Supplementary material for: Dietary Variation and Evolution of Gene Copy Number among Dog Breeds
Source: PLoS One. 2016 Feb 10;11(2):e0148899. doi: 10.1371/journal.pone.0148899 (PMC4749313; doi:10.1371/journal.pone.0148899)
Supplement: S8 Table — (PDF) [file pone.0148899.s012.pdf]

Table S8: Diploid *PHYH* copy number and technical error estimates from ddPCR

Breed	Sample	Diploid *PHYH* CN Estimate	Diploid *PHYH* CN	Poisson Max CN Estimate*	Poisson Min CN Estimate*
Alaskan malamute	AM-1	8.1	8	9.2	7.1
Alaskan malamute	AM-2	13.3	13	15	11.6
Alaskan malamute	AM-3	10.0	10	11.6	8.5
Alaskan malamute	AM-4	5.9	6	6.9	4.9
Alaskan malamute	AM-5	11.5	12	13.3	9.6
Alaskan malamute	AM-6	8.5	9	9.2	7.8
Alaskan malamute	AM-7	9.1	9	11.2	7
Alaskan malamute	AM-8	7.5	8	8.1	6.8
Alaskan malamute	AM-9	14.6	15	16	13.2
Alaskan malamute	AM-10	25.5	26	27	24.1
Alaskan malamute	AM-11	10.1	10	11	9.1
Alaskan malamute	AM-12	14.7	15	16.6	12.8
Alaskan malamute	AM-13	13.8	14	16.4	11.2
Shar Pei	CSP-1	24.0	24	30	18
Shar Pei	CSP-2	27.0	27	34	20
Shar Pei	CSP-3	13.4	13	17.4	9.5
Shar Pei	CSP-4	25.2	25	29.9	20.4
Shar Pei	CSP-5	17.1	17	21.3	12.8
Shar Pei	CSP-6	25.0	25	56	16
Shar Pei	CSP-7	14.7	15	18.1	11.4
Shar Pei	CSP-8	8.1	8	10.1	6
Japanese	AK-1	13.1	13	14.1	12.1
Japanese	SI-1	16.9	17	20.7	13.1
Japanese	SI-2	18.0	18	24	13
Japanese	SI-3	16.4	16	18.3	14.4
Japanese	SI-4	13.9	14	18.3	9.5
Japanese	AK-2	31.5	32	35	27.9
Japanese	AK-3	24.7	25	26.3	23
Japanese	AK-4	22.0	22	23	20.7
Japanese	AK-5	32.1	32	33.9	30.2
Japanese	AK-6	29.3	29	32.8	25.9
Japanese	AK-7	34.4	34	37.9	30.9
Pekingese	PK-1	16.5	17	17.8	15.3
Pekingese	PK-2	13.6	14	14.4	12.8
Pekingese	PK-3	14.5	15	16.5	12.4
Pekingese	PK-4	16.5	17	17.6	15.4
Pekingese	PK-5	11.4	11	12.7	10.2
Pekingese	PK-6	9.5	10	11.2	7.8
Pekingese	PK-7	9.5	10	10.5	8.5

Pekingese	PK-8	9.8	10	11.1	8.5
Pekingese	PK-9	13.2	13	14.9	11.4
Pekingese	PK-10	11.1	11	13.2	9
Pekingese	PK-11	14.1	14	14.9	13.3
Pekingese	PK-12	17.2	17	20.4	14
Pekingese	PK-13	16.0	16	23	10
Pekingese	PK-14	11.2	11	12.1	10.3
Pekingese	PK-15	18.5	19	21.5	15.6
Siberian husky	SH-1	14.6	15	16	13.2
Siberian husky	SH-2	15.8	16	17.3	14.3
Siberian husky	SH-3	13.3	13	14.6	11.9
Siberian husky	SH-4	14.8	15	15.7	13.8
Siberian husky	SH-5	16.3	16	17.5	15
Siberian husky	SH-6	14.3	14	15.6	12.9
Siberian husky	SH-7	9.3	9	10.2	8.5
Siberian husky	SH-8	14.7	15	15.9	13.5
Siberian husky	SH-9	18.0	18	20.6	15.4
Siberian husky	SH-10	20.5	21	22.6	18.3
Siberian husky	SH-11	20.9	21	23.1	18.7
Siberian husky	SH-12	10.5	11	11.4	9.5
Siberian husky	SH-13	21.8	22	23.3	20.2
Siberian husky	SH-14	12.3	12	13	11.5
Siberian husky	SH-15	11.5	12	12.2	10.8

*Droplet Digital PCR generates maximum and minimum technical error estimates from one replicate.
