# Supplementary material for: Dietary Variation and Evolution of Gene Copy Number among Dog Breeds
Source: PLoS One. 2016 Feb 10;11(2):e0148899. doi: 10.1371/journal.pone.0148899 (PMC4749313; doi:10.1371/journal.pone.0148899)
Supplement: S9 Table — (PDF) [file pone.0148899.s013.pdf]

Table S9. Primer and Probe sequences used for ddPCR assays for target and reference genes.

Target Gene	Forward Primer	Reverse Primer	Probe	Reference Gene	Forward Primer	Reverse Primer	Probe
AMY2B	ccaaacctggacggacatct	tatcgttcgcattcaagagcaa	(FAM/MGB) tttgagtggcgctgg	*C7orf28B*	ttgtgcaggatcagagcatc	caacacaggttgaccaagga	(VIC/TAMRA) tgccatttgtgtgca tcccc
GCKR	gctctaccctcctctaatttccgtta	ttggaggatcttcccaagaagca	(MGB/VIC) taagcaccaagtggatact	*CFTR*	tttgcatactgtatctactttgttcctgtt	ctgattcactggataattatgagcctagt	(MGB/FAM) cagagctgcacctattc
PHYH	acagtaatgacatcatctgccaatttcat	gcaatggacacatctctcattatcatcat	(MGB/VIC) accagtttgaaaggatct	*CFTR*	tttgcatactgtatctactttgttcctgtt	ctgattcactggataattatgagcctagt	(MGB/FAM) cagagctgcacctattc
