# Supplementary material for: Dietary Variation and Evolution of Gene Copy Number among Dog Breeds
Source: PLoS One. 2016 Feb 10;11(2):e0148899. doi: 10.1371/journal.pone.0148899 (PMC4749313; doi:10.1371/journal.pone.0148899)
Supplement: S10 Table — (PDF) [file pone.0148899.s014.pdf]

Table S10. Considered coordinates and location of ancestry informative SNPs for *AMY2B*, *GCKR*, and *PHYH*.

Gene	Coordinates[1]	Expanded Coordinates[2]	ai.SNPs[3]
GCKR	chr17:24411781- 24887707	chr17:23911781-25387707	chr17.24309172<br>chr17.24312880<br>chr17.25295979
PHYH	chr2:25986659-26125955	chr2:25486659-26625955	chr2.25670153
AMY2B	chr6:49348429-50097961	chr6:48848429-50597961	chr6.50074785<br>chr6.50081582<br>chr.50089070

[1]These coordinates range from the transcription start site of the nearest non-overlapping 5' and 3' flanking genes according to the UCSC genome browser using the CanFam2 build.

[2]Consistent with Vonholdt et al. (2010), these coordinates expand the previous column by 500kb on each side to include the full potential range of an introgressed haplotype.

[3]ai.SNPs refer to ancestry informative SNPs. Defined by an absolute value ΔDAF between dogs and wolves greater than two standard deviations from the genome-wide mean.
